# Conformational Plasticity of HLA-B27 Molecules Correlates Inversely With Efficiency of Negative T Cell Selection

**DOI:** 10.3389/fimmu.2020.00179

**Published:** 2020-02-11

**Authors:** Bernhard Loll, Christine Rückert, Barbara Uchanska-Ziegler, Andreas Ziegler

**Affiliations:** ^1^Institut für Chemie und Biochemie, Abteilung Strukturbiochemie, Freie Universität Berlin, Berlin, Germany; ^2^Institut für Immungenetik, Charité - Universitätsmedizin Berlin, Freie Universität Berlin, Berlin, Germany; ^3^Ziegler Biosolutions, Waldshut-Tiengen, Germany

**Keywords:** HLA-B27, X-ray structure, peptide binding modes, conformational flexibility, T cell selection, central tolerance, molecular mimicry, ankylosing spondylitis

## Abstract

The development of autoimmune disorders is incompletely understood. Inefficient thymic T cell selection against self-peptides presented by major histocompatibility antigens (HLA in humans) may contribute to the emergence of auto-reactive effector cells, and molecular mimicry between foreign and self-peptides could promote T cell cross-reactivity. A pair of class I subtypes, HLA-B2705 and HLA-B2709, have previously been intensely studied, because they are distinguished from each other only by a single amino acid exchange at the floor of the peptide-binding groove, yet are differentially associated with the autoinflammatory disorder ankylosing spondylitis. Using X-ray crystallography in combination with ensemble refinement, we find that the non-disease-associated subtype HLA-B2709, when presenting the self-peptide pGR (RRRWHRWRL), exhibits elevated conformational dynamics, and the complex can also be recognized by T cells. Both features are not observed in case of the sequence-related self-peptide pVIPR (RRKWRRWHL) in complex with this subtype, and T cell cross-reactivity between pGR, pVIPR, and the viral peptide pLMP2 (RRRWRRLTV) is only rarely observed. The disease-associated subtype HLA-B2705, however, exhibits extensive conformational flexibility in case of the three complexes, all of which are also recognized by frequently occurring cross-reactive T cells. A comparison of the structural and dynamic properties of the six HLA-B27 complexes, together with their individual ability to interact with T cells, permits us to correlate the flexibility of HLA-B27 complexes with effector cell reactivity. The results suggest the existence of an inverse relationship between conformational plasticity of peptide-HLA-B27 complexes and the efficiency of negative selection of self-reactive cells within the thymus.

## Introduction

Although the vast majority of autoimmune diseases is associated with *HLA class II* alleles (1), the first association detected and still one of the strongest found so far is that between the class I gene HLA-B^*^27 and the autoinflammatory rheumatic disorder ankylosing spondylitis (AS) (2, 3). Rats and mice transgenic for HLA-B^*^27 demonstrate a direct involvement of the HLA-B27 protein in the development of diseases in the animals that share many features with AS (4–8). In humans, not all of the >170 alleles (“subtypes”) of HLA-B^*^27 are associated with AS: while the prototypical HLA-B^*^27:05 subtype (B^*^27:05 in short) is AS-associated, HLA-B^*^27:09 (in short, B^*^27:09) is not (9). Both alleles encode a heavy chain (HC) that associates non-covalently with a light chain, β_2_-microglobulin (β_2_m), and a self- or foreign peptide. The B2705 and B2709 complexes are distinguished only by an Asp116His exchange within their HC, located on the floor of the peptide binding groove within the F-pocket. Comparative studies of these two subtypes can thus be regarded as promising to understand various aspects of AS pathogenesis.

Functional, X-ray crystallographic, spectroscopic, and calorimetric experiments as well as molecular dynamics (MD) simulations have already shown that the Asp116His micropolymorphism of the HC, combined with differential peptide binding, leads to an overall structural polymorphism of the subtypes that is responsible for several distinct features of B2705 and B2709 [reviewed by (10–12)]. For example, cytotoxic T lymphocytes (CTL) directed against the self-peptides pVIPR [RRKWRRWHL, derived from vasoactive intestinal peptide type 1 receptor (residues 400–408)] and pGR [RRRWHRWRL, derived from glucagon receptor (residues 412–420)] have been found in individuals with B^*^27:05 (13, 14). About one sixth of these T cells cross-reacts with the viral pLMP2 peptide [RRRWRRLTV, derived from latent membrane protein 2 (residues 236–244)] of Epstein-Barr virus (EBV) (15), suggesting the existence of B2705-restricted molecular mimicry between the three peptides (14). In contrast, CTL cross-reactivity between the pVIPR and pLMP2 peptides has only rarely been observed when these ligands are displayed by B2709, suggesting lack of molecular mimicry (16). X-ray crystallographic studies support these conclusions (15) and indicate that structural similarity does not only form the basis for the observed CTL cross-reactivity in B^*^27:05^+^ individuals, but argues also in favor of a relationship with the pathogenesis of AS. Although crystallographic studies had thus implicated structural features of complexes with the pGR, pVIPR, and pLMP2 peptides displayed by B2705 as the basis for CTL recognition and suggested a connection with the initiation of AS, results from infrared (IR) spectroscopy demonstrated that an increase in conformational flexibility might in fact be more important than structural peculiarities in explaining the association of certain HLA-B^*^27 subtypes (besides B^*^27:05 also B^*^27:04) with disease (17).

In the present work, we extend the structural analyses of complexes with pGR and related peptides to the B2709 subtype, taking functional studies with CTL from B^*^27:09^+^ individuals into account (16). We address three issues in particular: (i) Is the extent of peptide dynamics a consequence of display by a particular HLA-B27 subtype? (ii) Does the overall conformational plasticity of a given peptide-HLA-B27 complex allow to correlate this property with CTL reactivity? and (iii) Do the complexes of B2709 displaying pGR, pVIPR, or pLMP2 exhibit a lack of comprehensive molecular mimicry? Together with previously published studies (13–16, 18) the high resolution structure of pGR-B2709 that we describe here permits to shed light on these questions and indicates that the occurrence of peripheral self-reactive CTL is connected with the conformational plasticity of peptide-HLA-B27 complexes.

## Materials and Methods

### Protein Preparation, Crystallization and Data Collection

The peptide pGR (RRRWHRWRL) was purchased from Alta Bioscience (Birmingham, UK). B2709 HC, and β_2_m were expressed separately as inclusion bodies in *Escherichia coli*, dissolved in 50% urea and pGR-B2709 complexes were reconstituted using a protocol described previously (13, 19). The complexes were isolated by size exclusion chromatography and used for crystallization at concentrations of 13–15 mg/ml in 20 mM Tris/HCl (pH 7.5), 150 mM NaCl, 0.01% sodium azide. Crystals were obtained from drops mixed of 1.5 μl protein and 1.5 μl precipitant solution [12–16% (w/v) polyethylene glycol (PEG) 8000, 100 mM Tris/HCl, pH 7.5 or 8.0] in a hanging-drop vapor diffusion setup applying streak seeding techniques. Synchrotron X-ray diffraction data to 1.12 Å resolution were collected at European Synchrotron Radiation Facility (ESRF, Grenoble, France) at beamline ID 14-2 from cryo-cooled crystals with 10% (w/v) glycerol and 20% (w/v) PEG 8000 as cryoprotectant at 100 K. The data were processed and scaled with the HKL package (20) (Table 1). The raw diffraction images are made public through www.proteindiffraction.org (doi: 10.18430/m33czf).

**Table 1:** Data collection and refinement statistics.

**Data collection**	**pGR-B2709**
PDB entry	3CZF
Wavelength (Å)	0.933
Space group	*P*2_1_
Unit cell [*a,b,c* (Å); β (°)]	51.0, 81.9, 65.4; 108.9
Resolution (Å)^a^	30.0–1.1 (1.12–1.10)
Unique reflections^a^	196,702 (9,285)
Completeness (%)^a^	95.8 (90.4)
< I/(σ)>^a^	20.8 (2.7)
$R_{\text{sym}}^{\text{a},\text{b}}$	4.3 (28.8)
${\text{CC}}_{1/2}^{\text{a}}$	99.9 (80.7)
Wilson B factor (Å^2^)	14.7
**Refinement**	
Resolution (Å)	15.0–1.20
Number of reflections	149,186
Non-hydrogen atoms	4,243
$R_{\text{cryst}}^{\text{a},\text{c}}$	12.9 (13.8)
$R_{\text{free}}^{\text{a},\text{d}}$	14.9 (15.9)
Heavy chain, no. of atoms/average B factor (Å^2^)	2,439/12.5
β_2_m, no. of atoms/average B factor (Å^2^)	913/14.2
Peptide, no. of atoms/average B factor (Å^2^)	149/12.2
Water, no. of molecules/average B factor (Å^2^)	712/27.1
Glycerol, no. of atoms/average B factor (Å^2^)	30/23.4
rmsd^e^ from ideal geometry, bond length (Å)	0.011
bond angles (°)	1.427
Ramachandran outliers (%)	0.3
Ramachandran favored (%)	98.1

a *Values in parentheses refer to the highest resolution shell*.

*^b^R_sym_ = Σ_h_Σ_i_ | I_h, I_- < I_h_>| / Σ_h_Σ_i_I_h, i_*.

*^c^R_cryst_ = Σ_h_ | F_o_-F_c_ | / Σ F_o_ (working set, no σ cut-off applied)*.

*^d^R_free_ is the same as R_cryst_, but calculated on 2.5% of the data excluded from refinement*.

e *Root-mean-square deviation (rmsd) from target geometries*.

### Structure Determination, Refinement, and Analysis

The structure of pGR-B2709 was solved by molecular replacement with the program EPMR (21) using water- and peptide-depleted m9-B2709 structures as search model (PDB entry 1K5N) (22). The crystallographic asymmetric unit contains one pGR-B2709 complex with a Matthews coefficient of 2.8 Å^3^ Da^−1^ corresponding to a solvent content of 55% (23). For calculation of a free *R*-factor, a randomly generated set of 2.5% of the reflections from the diffraction data set was used and excluded from refinement. Restrained maximum-likelihood refinement was performed using REFMAC5 (24) including anisotropic B-factor refinement followed by iterative manual model building with the program COOT (25). Water molecules were positioned with ARP/wARP (26) and manually inspected. For data collection and refinement statistics see Table 1. Intermediate and final structures were evaluated with MOLPROBITY (27) and PROCHECK (28). Figures were prepared with PYMOL (29). The atomic coordinates and structure amplitudes have been deposited in the Protein Data Bank (PDB entry 3CZF).

### Ensemble Refinement

The starting structures for ensemble refinements as implemented in PHENIX (30) were prepared as described (18, 31). Apart from pGR-B2709 (this study), the following complexes were analyzed: pGR-B2705 [PDB entry 2A83 (14)], pVIPR-B2709 (PDB entry 5IB5), pVIPR-B2705 [PDB entry 5IB2 (18)], pLMP2-B2709 [PDB entry 1UXW (15)], and pLMP2-B2705 [PDB entry 1UXS (15)]. Briefly, alternate conformations of amino acid side chains were removed from the deposited structures in the PDB and the occupancies adjusted to 1. Prior to refinement, explicit hydrogen atoms were generated with phenix.ready_set. The ensemble refinements were executed with the standard settings. For glycerol molecules, harmonic restraints were set to avoid stochastic displacement during simulations.

## Results

### Structural Characteristics of the pGR-B2709 Complex

pGR-B2709 crystallized in space group *P*2_1_ like B2705 bound to pGR (14), or both subtypes in complex with the sequence-related pVIPR (18) and pLMP2 peptides (15). The structure of the pGR-B2709 complex exhibited very high resolution of 1.2 Å and was refined to R/R_free_ of 0.129/0.149. For detailed X-ray diffraction and refinement statistics see Table 1. As expected, the single polypeptide chains, HC and β_2_m, show the protein fold characteristic for HLA class I molecules (32, 33). Differences to other HLA-B27 complexes are restricted to polymorphic residues such as Asp116His in the B2705/B2709 pair, to conformations of solvent-exposed amino acid side-chains and peptide residues, as well as to the location of water molecules. Notably, none of the peptide residues in pGR-B2705 or pGR-B2709 is involved in crystal contacts.

**Figure 1:**
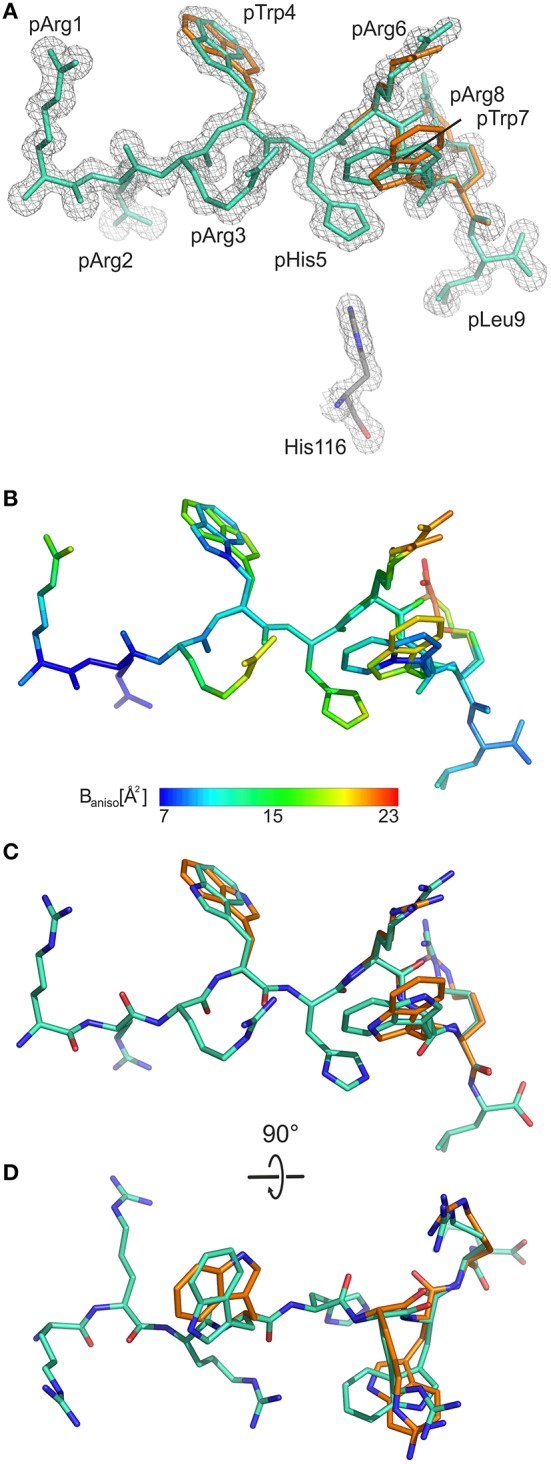
General structural properties of the bound pGR peptide displayed by B2709. For sake of clarity, water molecules are omitted in all representations. In **(A–C)**, the view is from the side of the α2-helix, which is not displayed. **(A)** Final 2F_o_-F_c_ electron density maps (blue mesh) contoured at 1.3 σ, with pGR in A-conformation (cyan) and in B-conformation (orange) shown in stick representation. The subtype-specific residue His116 of the HC is shown as well. **(B)** Color scheme depicting the anisotropic B-factor distribution in both pGR conformations. **(C)** Superimposition of both pGR conformations, viewed as in **(A)**. **(D)** Superimposition of both pGR conformations, 90° rotated toward the viewer in comparison to **(C)**.

As the calculated electron density map is of excellent quality, two conformations of the pGR Cα-backbone, designated A and B, could readily be assigned, between the two arginine residues at position 6 (p6) and p8 (Figure 1A), although the overall difference exhibited by both peptide Cα-traces is minimal [root mean square deviation (r.m.s.d.) of 0.2 Å]. From the electron density, the ratio of conformations A to B was estimated to be about 70–30%. The B-factors of the bound peptides in the A and the B conformation are virtually identical (Figure 1B). In addition to the difference between the Cα-backbones of the peptide, double conformations are also observed for the side chains of four peptide residues, pTrp4, pArg6, pTrp7, and pArg8 (Figures 1C,D).

### Comparative Structural Features of pGR-HLA-B27 Complexes

When the nona-peptides are neglected, the structures of pGR-B2709 and pGR-B2705 superimpose with an r.m.s.d. of 0.3 Å for the Cα-atoms and must thus be considered virtually identical. Both pGR peptide conformations assume the “p6α” binding mode (i.e., main-chain ϕ/ψ torsion angles in α-helical conformation at p6) in the B2709 subtype. This unconventional (non-canonical, NC) conformation had been detected so far in case of B2705 in complex with the peptides pVIPR (18), pLMP2 (15), pGR (14), and in the pVIPR-U5-B2709 [modified pVIPR with citrulline at p5 (34)] as well as in the pVIPR-B2706 (17) complex. Whereas, easily distinguishable differences between the Cα-conformations of pArg3 to pArg8 were observed in pGR-B2705 (r.m.s.d. of 0.5 Å), the differences in pGR-B2709 are very small and limited to the region from pArg6 to pArg8. A comparison of the different conformations of the pGR peptide in B2709 with those in B2705 reveals that they are slightly more similar to pGR-B2705-B (r.m.s.d. for Cα positions of 0.2 Å) than to pGR-B2705-A (r.m.s.d. for Cα positions of 0.4 Å).

**Figure 2:**
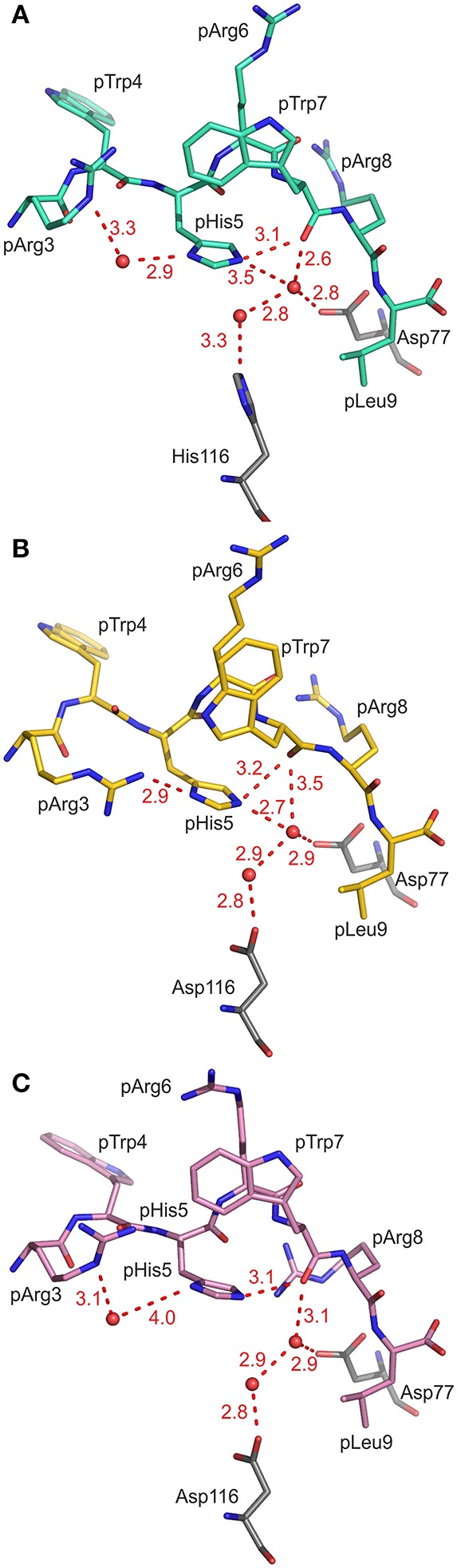
Contacts of the peptide residue pHis5. Peptide residues p3 to p9 and the HC residues Asp77 and His/Asp116 are shown in stick representation, water molecules are drawn as red spheres, hydrogen bonds are depicted as red dashed lines, and all distances are given in Å. The view is through the α2-helix (removed), roughly along the length of the peptide binding groove toward the N-terminal peptide residues. **(A)** pHis5 of pGR-B2709 in A-conformation (cyan) is embedded in a dense hydrogen bonding network resulting in direct and indirect contacts. **(B)** pHis5 of pGR-B2705 in A-conformation (yellow) is involved in hydrogen bonding which includes several contacts that are mediated by water molecules. **(C)** pHis5 of pGR-B2705 in B-conformation (pink) is involved in fewer contacts compared to **(A,B)**.

As a consequence of the pGR peptide's NC binding mode in complex with B2709, the side chain of pHis5 points toward the interior of the binding groove (Figures 1, 2A) and is embedded in a dense network of hydrogen bonds which, however, do not directly contact the subtype-specific His116 residue. pHis5 adopts a single conformation, in contrast to the double conformation observed for this residue in pGR-B2705-A and -B (Figures 2B,C) and the above-mentioned four other peptide residues. In B2709, pHis5 engages in a direct contact to the backbone carbonyl of pTrp7 and an indirect water-mediated contact to pArg3NE as well as a further indirect interaction to HC residue Asp77 which is located on the α1-helix (Figure 2A). A detailed comparison with the contacts of pHis5 in the B2705 subtype (Figures 2B,C) demonstrates that the pattern of interactions in B2709 is a “hybrid” between the contacts exhibited by pHis5 in the two B2705 conformations. In B2709, the contacts to Asp77 and to His116 are more similar to those found in pGR-B2705-A (Figure 2B), while the interaction with pArg3 resembles that observed in pGR-B2705-B (Figure 2C). Table 2 provides a detailed account of the contacts of the pGR peptide in complex with B2705 and B2709. Of note, the van der Waals contact between pTrp4 and the HC residue Ile66 is present in the complexes of both subtypes. In contrast, this contact is restricted to B2705 presenting pVIPR or pLMP2 (13, 15, 18), suggesting that the NC peptide binding mode could be a prerequisite for this interaction.

**Table 2:** Comparison of pGR conformations in the B2705 and B2709 subtypes.

	**pGR-B2705 conformations A and B**				**pGR-B2709 conformations A and B**			
	**Peptide residue**	**Contact residue**	**Distance**	**Interaction**	**Peptide residue**	**Contact residue**	**Distance**	**Interaction**
**p1 & p2**	Contacts formed by Arg1 and Arg2 are very similar in both complexes; the side chain of Arg1 is solvent-exposed, whereas Arg2 is buried							
**p3**	pArg3^N^	Tyr99^OH^	3.09	HB	pArg3^N^	Tyr99^OH^	3.1	HB
	pArg3	Tyr99, Leu156, Tyr159	3.6–4.0	vdW	pArg3	Tyr99, Leu156, Tyr159	3.6–4.0	vdW
	pArg3^NH2^(A)	pHis5^ND1^(A)	2.9	HB	pArg3^NE^	pTrp4^O^	2.8	HB
**p4**	The side chain of this residue is solvent-exposed in all complexes							
	pTrp4	Gln65, Arg62, Ile66	3.6-4.0	vdW	pTrp4	Gln65, Arg62, Ile66	3.6-4.0	vdW
**p5**	The side chain of this residue is buried in all complexes							
	pHis5^ND1^(A)	pArg3^NH2^(A)	2.9	HB	pHis5^NE2^	Lys70	3.5	HB
	pHis5^NE2^(B)	pTrp7^O^(B)	3.1	HB	pHis5^NE2^	pTrp7^O^(A)	3.1	HB
	pHis5^O^ (B)	pArg8^NH2^ (B)	3.5	HB	pHis5^NE2^	pTrp7^O^(B)	3.7	HB
**p6**	The side chain of this residue is solvent-exposed in all complexes							
	pArg6^O^(A)	pArg8^NH1^ (A)	2.8	HB	pArg6^O^(B)	pArg8^NH1^ (A)	2.8	HB
	pArg6^NH2^ (B)	Gln155^OE1^	2.8	HB	pArg6^O^(B)	pArg8^NH1^ (B)	2.8	HB
**p7**	pTrp7^O^(A)	pHis5^NE2^ (A)	3.3	HB	pTrp7	Val152, Trp147	3.6-4.0	vdW
	pTrp7^O^(B)	pHis5^NE2^ (B)	3.1	HB				
	pTrp7	Ala150, Val152	~3.6	vdW				
**p8**	The side chain of this residue is solvent-exposed in all complexes							
	pArg8^NE^ (A)	Glu76^OE1^	3.0	SB	Arg8^NE^ (A)	Glu76^OE2^ (A)	3.0	SB
	pArg8^NH2^ (A)	Thr73^OG1^	3.5	HB	Arg8^NE^ (B)	Glu76^OE2^ (A)	2.5	SB
	pArg8^NH2^ (B)	Asp77^OD2^	3.4	SB	Arg8^O^	Trp147^NE1^	2.9	HB
	pArg8^NE^ (B)	Asp77^OD1^	3.2	SB				
	pArg8^O^	Trp147^NE1^	2.9	HB				
**p9**	The side chain of this residue is buried in both complexes.							

*Distances are given in (Å). Only direct intra-peptide contacts and contacts between the peptides and HC residues [distance cut-off 3.5 Å; hydrogen bond (HB), salt bridge (SB)] are included; solvent-mediated interactions are omitted, and van der Waals (vdW) contacts are not given explicitly. Double conformations of amino acid residues are indicated by (A) or (B)*.

**Figure 3:**
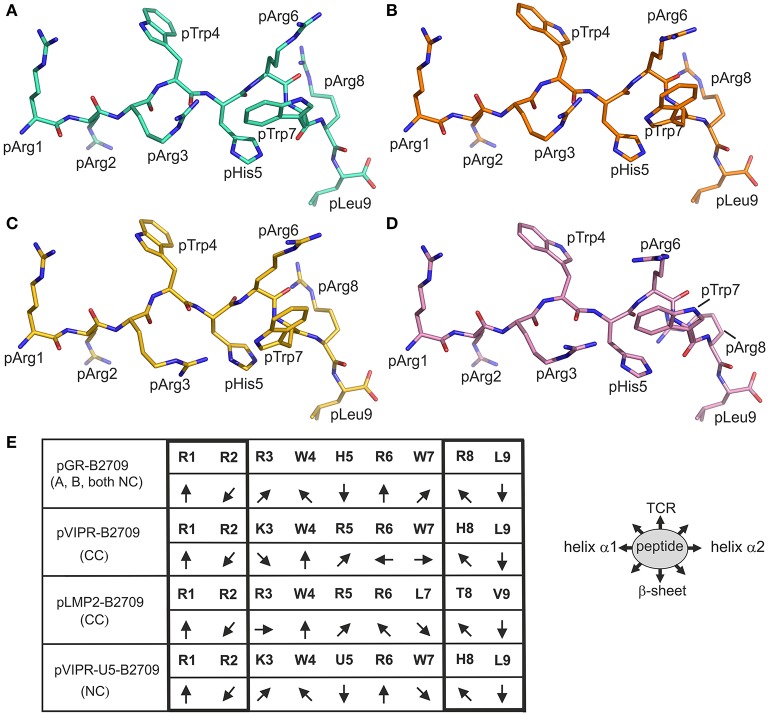
Comparison of the binding modes of pGR and three other peptides to B2709 and B2705. The peptides are viewed from the side of the α2-helix (removed). The representations resemble those in Figures 1A,C. **(A)** A-conformation of pGR-B2709 (cyan), **(B)** B-conformation of pGR-B2709 (orange), **(C)** A-conformation of pGR-B2705 (gold), **(D)** B-conformation of pGR-B2705 (pink). **(E)** Schematic representation of peptide side chain orientations when presented by B2709. The peptides are viewed from the N- to the C-terminus. The following ligands are shown: pGR (A- or B-conformation, both in NC binding mode), pVIPR (in CC binding mode), pLMP2 (in CC conformation), and pVIPR-U5 (pArg5 replaced by citrulline) in NC binding mode. The orientations of the peptide side chains are indicated and the primary sequence of the peptides is given. The right panel shows schematically the floor of the peptide binding groove indicated by “β-sheet” and binding region for a TCR by “TCR”.

The “hybrid” nature of structural features within the pGR-B2709 complex extends to properties of the side chains of peptide residues that exhibit conformational dimorphism. A comparison with the respective pGR-B2705-A and -B conformations (Figure 3) shows that pTrp4, pArg6, and pArg8 of pGR-B2709-A exhibit a nearly identical conformation to that found in B2705. In contrast, pTrp7 of pGR-B2709-A is very similar to the B conformation, which this residue adopts in pGR-B2705. In case of the B conformation in B2709, pTrp4 is identically and pArg6 similarly bound as in pGR-B2705-B. Conversely, pTrp7 and pArg8 in pGR-B2709-B are nearly identical to the A conformation, which is observed in B2705 (14).

Despite these differences, the results demonstrate that there are several features which are shared by the two pGR-HLA-B27 complexes. Irrespective of the subtype or the A or B conformations, the pGR residues pArg1 and pArg2 are identically anchored in the characteristic binding mode that has been observed before (13–15, 34–36), pHis5 is always nearly identically located, and the terminal pLeu9 is embedded within the F-pocket as in case of the peptides pVIPR (13), TIS (36), and pVIPR-U5 (34).

### Structural Similarities and Differences Between the pGR, pVIPR, and pLMP2 Peptides in Complex With HLA-B27 Subtypes

The isomorphous crystallization modes of the HLA-B27 complexes with the peptides pVIPR (18), pLMP2 (15), and pGR [(14) and this study], permit a detailed comparison of their structures. Previous work had already shown that the structures of these three peptides are very similar, at least in their N-terminal two thirds, when presented by the B2705 subtype, thereby furthering molecular mimicry (14). This is due to the NC binding mode of the displayed peptide which is exhibited by all of the complexes. In contrast, despite sharing the canonical conformation (CC), pVIPR and pLMP2 are differently presented by the B^*^2709 subtype, primarily because the side chains at p3 (Lys or Arg) and pArg6 point to distinct directions (Figure 3E) (15).

The comparison of the newly determined pGR-B2709 structure with those of pVIPR and pLMP2 reveals that pGR is presented by this subtype in a further distinct manner, which is dictated by the presence of the NC conformation (Figures 1–3). Despite the expected close similarity around the p1 and p2 as well as around the p8 and p9 peptide residues, the three peptides deviate considerably from p3 to p7 (Figure 3E). The most divergent conformations are exhibited by pTrp4 and pArg6 and the residue (either a tryptophan or a leucine) at p7 (Figure 3C). On the basis of these results, the presence of molecular mimicry between pGR, pVIPR, and pLMP2 when displayed by B2709 must be regarded as highly unlikely.

**Figure 4:**
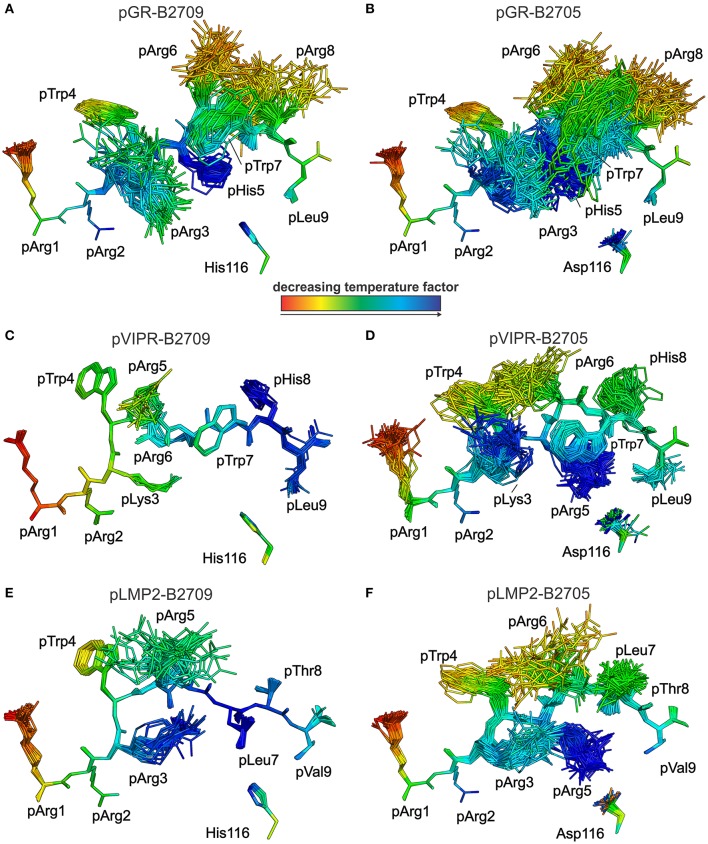
Results of the ensemble refinement performed for the structures of pGR, pVIPR, and pLMP2 bound to B2709 and B2705, respectively. For clarity, the HC and β_2_m are not shown. The peptide and the polymorphic residue at HC position 116 are displayed in stick representation. Peptides are color-coded by decreasing temperature factor from red to blue. In some structures, certain residues are partly “hidden” by others (e.g., pArg6 is “behind” pArg5 in the pLMP2-B2709 complex). **(A)** pGR-B2709, **(B)** pGR-B2705, **(C)** pVIPR-B2709, **(D)** pVIPR-B2705, **(E)** pLMP2- B2709, and **(F)** pLMP2- B2705. In the case of the pGR complexes, the A- and B-conformations of the peptide (always in NC binding mode) are depicted for each subtype, while the other peptides [including pVIPR (18)] are shown in a single conformation (either CC, pVIPR and pLMP2 bound to B2709, or NC, pVIPR and pLMP2 displayed by B2705).

**Figure 5:**
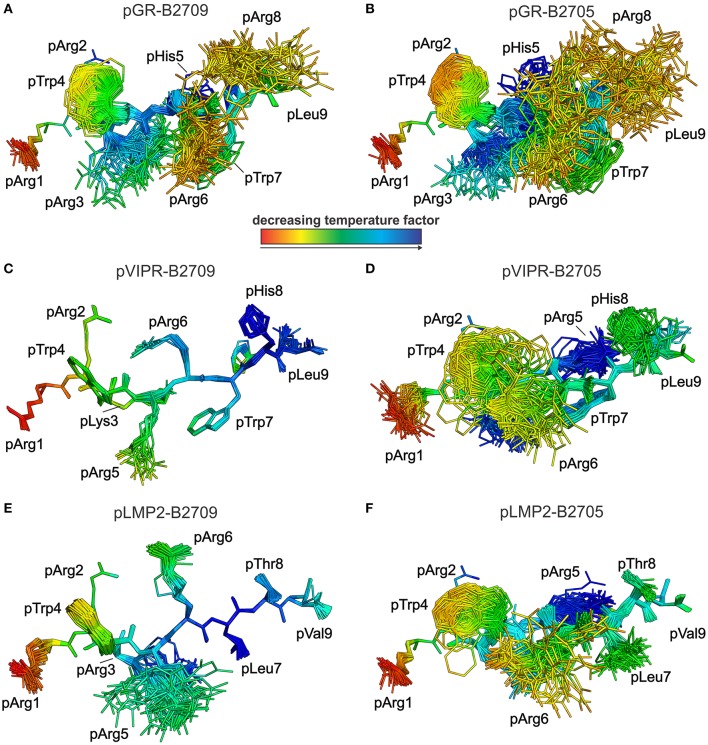
View as in Figure 4, but rotated by 90°. Peptides are oriented such as an approaching TCR would “see” them. They are color-coded by decreasing temperature factor from red to blue. **(A)** pGR-B2709, **(B)** pGR-B2705, **(C)** pVIPR-B2709, **(D)** pVIPR-B2705, **(E)** pLMP2-B2709, and **(F)** pLMP2-B2705. In some structures, certain residues are partly “hidden” by others (e.g., pArg2 is “behind” pTrp4 in the pGR-B2709 complex). For the conformations and binding modes of the peptides, please refer to the legend of Figure 4.

This conclusion is further supported by analyses of peptide plasticity using ensemble refinements (18, 31, 37, 38). This approach permits to gain information on dynamic properties of a protein whose structure had been solved by X-ray crystallography, by employing short, steered molecular dynamics (MD) simulations during the refinement process. The results (Figures 4, 5A,B) demonstrate that pArg2 and pLeu9 are virtually immobile, as expected for residues anchoring the pGR peptide within the binding groove. In contrast, not only the residues exhibiting double conformations, but all residues from pLys3 to pArg8 are characterized by extensive conformational plasticity in the complexes of both HLA-B27 subtypes presenting this peptide. The graphic presentation of ensemble-refined structures poses a problem not encountered when B-factors (temperature or Debye-Waller factors) calculated for conventionally refined structures (compare Figure 1B) are depicted. Since ensemble refinement allows to visualize B-factors independent from the atomic fluctuations that can be represented by multiple structures within the ensemble, high (low) B-factors do not necessarily reflect elevated (decreased) plasticity of residues within a molecule. This is due to the well-known fact that B-factors do not only report on atomic fluctuations, but are also influenced by factors such as crystal lattice defects, rigid body motions, occupancy levels, radiation damage, or refinement artifacts (39, 40). Consequently, regions with a greater conformational flexibility, as revealed by ensemble refinement, could have lower B-factors, since the ensemble is more precisely reflecting the electron density (41), as seen e.g., in the case of pArg3 in pLMP2-B2709 (Figure 4E) and pArg5 in pLMP2-B2705 (Figure 4F). On the other hand, in case of pArg1 the conformational flexibility is restricted due to π-π interactions with the side chains of Arg62 and Trp167 of the heavy chain (Figures 4, 5), that are both solvent exposed. The protein environment could thus reduce the number of possible side chain conformations. In contrast, the anchoring residue pArg2 displays lower B-factors (Figures 4, 5), since it is not pointing toward the solvent but into the interior of the protein and as described for pArg1, the number of side chain conformations is limited due to interactions with residues of the heavy chain.

As previously described by us in detail (18), the pVIPR peptide is considerably more mobile in B2705 than in B2709. Only pArg5 shows a moderate degree of plasticity in B2709 (Figures 4, 5C). In the case of the pLMP2 peptide, the difference between the subtypes is not as pronounced as for pVIPR, but the peptide is definitely more mobile in its middle (residues p3–p7) when bound to B2705 (compare Figures 4, 5E,F).

With regard to molecular mimicry, the ensemble refinements permit to conclude that the probability that a TCR would encounter a similar collection of structures among the three peptides is far greater in the B2705 subtype than in the case of B2709. For example, when comparing pVIPR-B2709 and pLMP2-B2709, only the area “above” pArg1, pTrp4, and pArg5 might evoke a response by a cross-reactive CTL, and an extension of this cross-reactivity to pGR-B2709 is difficult to imagine (compare Figures 4, 5A,C,E). This is very different in the case of the disease-associated subtype (compare Figures 4, 5B,D,F). The ensemble of structures exhibited by peptides bound to B2705 that is available for CTL recognition is much larger than in case of the B2709 subtype.

### Distinct Dynamic Properties of the Peptide Binding Grooves of the B2705 and B2709 Subtypes

**Figure 6:**
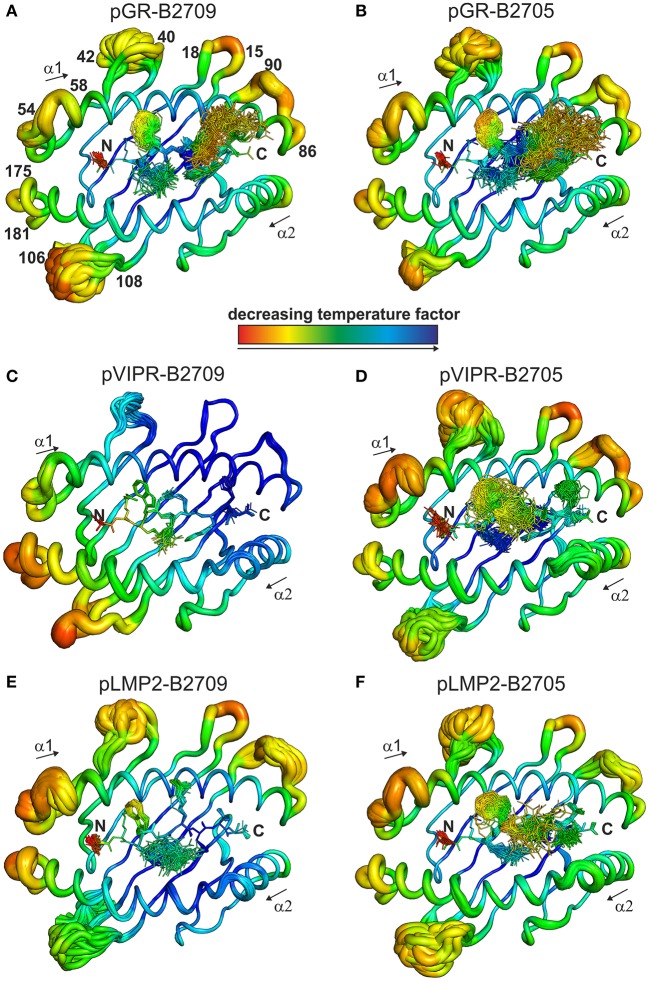
View onto the peptide binding groove, comparable to Figure 5. In addition to the peptide in stick representation, the HC in coil representation is shown. For clarity, the α3 domain of the HC and β_2_m are not displayed. Color-coding by decreasing temperature factor from red to blue and in addition the diameter of the coil reflect the mobility: the larger the diameter, the higher the mobility. Numbers indicate the position of amino acids within the protein sequence of the HC. **(A)** pGR-B2709, **(B)** pGR-B2705, **(C)** pVIPR-B2709, **(D)** pVIPR-B2705, **(E)** pLMP2-B2709, and **(F)** pLMP2-B2705.

The fact that the conformational plasticity of the three peptides depends on the HLA-B27 subtype by which they are displayed, leads to the question how the hidden Asp116His micropolymorphism in the F pocket can influence the flexibility of peptide residues that may be separated by more than 20 Å as in the case of His116 and the guanidinium group of pArg1 (compare e.g., Figures 4, 5C,D). Therefore, we analyzed also the overall appearance of the binding grooves by ensemble refinement. Figure 6 depicts the six complexes recognizable by a TCR. It is immediately obvious that pVIPR-B2709 (Figure 6C) exhibits a greatly diminished degree of mobility, both of the peptide and of large parts of the binding groove. This property is not exhibited by any of the other complexes. In particular, the F pocket, which accommodates the polymorphic residue 116 (His in B2709), the peptide's C-terminus, and its surroundings show very little flexibility. A comparison with the other five complexes reveals that large parts of the α1-helix and the loops “below” it (HC residues 13–18 and 86–90, Figure 6) as well as the beginning of the α2-helix exhibit very little dynamics. Only that section of the binding groove accommodating the N-terminal third of pVIPR as well as the loop comprising residues 106–108 show a limited degree of flexibility. Consequently, the three B2709 complexes share neither a pronounced structural nor a dynamic similarity, in contrast to the three B2705 complexes (Figures 6B,D,F). Although the ensemble refinements of the peptide-HLA-B27 complexes provide a fairly good idea of the differential dynamics which characterizes each of the binding grooves, the results do not give a hint for the reasons underlying F-pocket effects on the flexibility of distant peptide residues (see the interaction between the guanidinium moiety of pArg1 and Asp/His116 mentioned above).

## Discussion

Conformational plasticity is a prerequisite for the successful interaction of MHC molecules with extra- and intracellular binding partners. Following the pioneering studies with peptide-HLA-A2 complexes and TCR molecules (42–44), also HLA-B27 subtypes have been employed in experimental studies regarding the influence of a micropolymorphism in the F-pocket on the dynamics of the peptide as well as the HC (17, 45–48). These studies proved, that HC dynamics is more pronounced in B2705 than in B2709, irrespective of the bound peptide. The Asp116His polymorphism is thus not only linked to AS, but also to HC flexibility. MD simulations support these results and further suggest that HC dynamics and the conformational plasticity of natural peptide ligands influence each other (45, 47, 49–52).

Although all MD simulations performed in the context of these studies indicate a higher flexibility of the B2705 F-pocket, one could argue that they examine rather limited time spans, “several orders of magnitude shorter than more biologically relevant timescales,” as pointed out by Buckle and Borg (53), indicating a need for additional dynamics-directed experimental studies. The present results are based on a complex of B2709 and the pGR peptide solved at near-atomic resolution, allowing to investigate the structure in unprecedented detail. They permit an in-depth comparison with pGR-B2705 [solved at 1.50 Å resolution; (14)], relying on conventional crystallographic procedures (Figures 1–3 and Table 2), but also on the application of ensemble refinement to obtain an estimate of the conformational plasticity of the molecules under investigation (18, 31, 37, 38, 53).

While the two complexes displaying pGR are characterized by similar peptide dynamics (Figures 4, 5A,B), this property is much less pronounced, though still relatively similar in complexes with the viral pLMP2 peptide (Figures 4, 5E,F). However, a different situation is evident in the case of the pVIPR peptide: while peptide plasticity is nearly absent when pVIPR binds to B2709 (Figures 4, 5C), the ligand exhibits drastically enhanced mobility when bound to B2705 (Figures 4, 5D). This elevated plasticity does not depend on a dual peptide conformation as originally described by us (13). pVIPR is in fact normally displayed by B2705 in a single NC conformation, since the dual binding mode is induced by a metal ion bound to the peptide (18). In addition, ensemble refinement analyses show that enhanced flexibility is not only observed for peptides bound to the complexes, but characterizes also the HC (Figure 6). This HC flexibility is exhibited by residues belonging to the beginning of the α1- and the end of the α2-helices and the HC loops “beneath” the binding groove (Figure 6A). The pVIPR-B2709 complex, however, shows a different picture: while the regions comprising residues 54–58 and 175–181 (at the “left” end of the binding groove, opposite to each other), as well as 106–108 reveal conformational flexibility, the “right” part of the binding groove, “above” the F-pocket and within a distance of about 15 Å from His116, exhibits a nearly complete lack of mobility (Figure 6C). These results are in complete agreement with the IR spectroscopic studies of Fabian et al. which demonstrated that the B2705 HC is more flexible than that of B2709, irrespective of the bound peptide (47). All data are listed in Table 3, together with experimental results obtained previously with two further peptides, pVIPR-U5 (a citrullinated version of pVIPR, RRKWURWHL, U = citrulline) (34) and TIS (RRLPIFSRL), a proven self-peptide for both B2709 and B2705 (36). Experiments with another pair of differentially AS-associated subtypes, B2704 and B2706, show also that conformational flexibility characterizes AS-associated subtypes (17).

**Table 3:** Comparison of structural and dynamic properties of HLA-B27 complexes with their CTL-reactivity.

	**Non AS-associated**		**AS-associated**	
**Peptide**	**B2709**		**B2705**	
**pGR**	Peptide conformation	NC, double conformation	Peptide conformation	NC, double conformation (14)
	Peptide flexibility	High (ER)	Peptide flexibility	High (ER)
	HC flexibility	High (ER)	HC flexibility	High (ER)
	Presence of CTL	Yes (16)	Presence of CTL	Yes (14)
**pVIPR**	Peptide conformation	CC (18)	Peptide conformation	NC (18)
	Peptide flexibility	Marginal [ER, NMR (18)]	Peptide flexibility	High [ER, NMR (18)]
	HC flexibility	Marginal {ER, low [IR (47)]}	HC flexibility	High [ER, IR (47)]
	Presence of CTL	No^&^ (16, 54)	Presence of CTL	Yes (16, 54)
**pLMP2**	Peptide conformation	CC (15)	Peptide conformation	NC (15)
	Peptide flexibility	High [ER (18)]	Peptide flexibility	High [ER (18)]
	HC flexibility	Intermediate {ER, low [IR (48)]}	HC flexibility	High [ER, IR (46)]
	Presence of CTL^*^	Yes (16, 54)	Presence of CTL	Yes (16, 54)
**pVIPR-U5**	Peptide conformation	NC (34)	Peptide conformation	CC (34)
	Peptide flexibility	ND	Peptide flexibility	ND
	HC flexibility	Low [IR (48)]	HC flexibility	High [IR (48)]
	Presence of CTL	ND	Presence of CTL	ND
**TIS**	Peptide conformation	CC (36)	Peptide conformation	CC (36)
	Peptide flexibility	ND	Peptide flexibility	ND
	HC flexibility	Low [IR (46)]	HC flexibility	High [IR (46)]
	Presence of CTL	ND	Presence of CTL	ND
	**B2706**		**B2704**	
**pVIPR**	Peptide conformation	CC/NC double conformation (17)	Peptide conformation	CC (17)
	Peptide flexibility	ND	Peptide flexibility	ND
	HC flexibility	Low [IR (17)]	HC flexibility	High [IR (17)]
	Presence of CTL	ND	Presence of CTL	ND

& pLMP2/pVIPR-cross-reactive CTL are rarely found in B*27:09^+^ individuals.

* *The presence of CTL against pLMP2 is a consequence of infection with EBV*.

Structural and dynamic properties were determined by X-ray crystallography with ensemble refinement (ER), NMR, and infrared (IR) spectroscopy. CTL-reactivity is attributed to a given complex in individuals with the respective HLA-B^*^27 subtype (ND, not determined).

Collectively, the X-ray crystallographic and dynamic studies suggest that (i) the occurrence of peripheral CTL against a given peptide-HLA-B27 complex correlates with the presence of an elevated degree of conformational plasticity of the peptide binding groove; (ii) as a rule, this flexibility is connected with AS-associated subtypes such as B2705 (46–48) and B2704 (17), although exceptions do exist (compare pGR-B2709, Figures 4, 5, 6A and Table 3); (iii) Neither a double conformation nor an NC binding mode of a peptide are obligatorily connected with the occurrence of CTL and disease [Table 3; (16, 17)]. For example, pVIPR-U5 is bound by B2709 in an NC conformation, but the HC flexibility was found to be low, while the CC binding mode of this peptide to B2705 did not change the elevated HC plasticity (48).

The results presented in Table 3 imply that the Asp116His polymorphism within the F pocket is not only closely linked to the presence (B^*^27:05) or absence (B^*^27:09) of AS and other HLA-B^*^27-associated autoinflammatory diseases, but is also involved in determining the fate of self-reactive T cells in the thymus. Together with previously obtained functional data (16), our results establish a link between the flexibility of HLA class I complexes and the occurrence of CTL directed against self-peptidesat least in the two HLA-B27 subtypes investigated here: the efficiency of negative T cell selection within the thymic medulla is impaired when T cells encounter a highly flexible binding groove, such as found for pGR-B2709, pGR-B2705, and pVIPR-B2705 (Figures 6A,B,D and Table 3), and when peptide dynamics is elevated beyond a certain level. This level is currently ill-defined, but visual inspection as well as a comparison of the number of conformations observed for the side chain of a particular residue within an ensemble allow to conclude that a single flexible peptide residue (pArg5 in pVIPR-B2709, Figures 4, 5C) does not prevent efficient T cell selection. Plasticity of further positions, as in pVIPR-B2705 (Figures 4, 5D), on the other hand, leads to failure of negative selection against this ligand. Therefore, our results indicate the existence of a reciprocal relationship between the efficiency of negative selection within the thymus (55–57) and the extent of conformational plasticity of peptide-HLA-B27 complexes.

If this scenario is correct, why are the surviving CTL able to recognize their target outside of the thymus? Several explanations can be thought of Yin et al. (58), e.g., by assuming that different concentrations of the peptide in distinct tissues could influence presentation or recognition. Another hypothesis takes modifications of the target peptide into account, such as citrullination, which can alter the binding mode to HLA-B27 subtypes (pVIPR-U5 as opposed to pVIPR) and which is known to influence CTL recognition (34). Whether regulatory T cells that have escaped negative thymic selection (59) play also a role in initiating or propagating AS, must currently be regarded as a matter of speculation.

A final question which we attempted to address is to explain the abundance of cross-reactive CTL against displayed pGR, pVIPR, or pLMP2 in B^*^27:05^+^ individuals and the scarcity of such CTL in B^*^27:09^+^ individuals (16). Molecular mimicry has been proven to exist in the HLA-B27 as well as other contexts (60, 61) and may contribute also to the emergence of cross-reactive CTL in certain subtypes. It remains still unknown whether these cells do really play a decisive role in the development of HLA-B^*^27-associated diseases (62). We have already pointed out that comprehensive molecular mimicry is a hallmark of presentation by B2705 (14), and we show now that there is neither a structural nor a dynamic basis for molecular mimicry when the B2709 subtype displays the peptides pGR, pVIPR, and pLMP2 (Figures 4–6).

The primary aim of our contribution was to investigate the relationship of peptide- and binding groove-plasticity of HLA-B27 subtypes to the efficiency of negative T cell selection. Due to their distinct, peptide-independent conformational flexibility (17, 46–48), however, peptide-devoid HC/β_2_m complexes within a cell possess already the potential to differentially influence peptide-loading processes (63–67). This could be relevant for the subtype-dependent initiation of HLA-B^*^27-associated diseases. A deeper insight into the dynamics of these molecules is thus crucial for a more detailed understanding of their function and interaction with other proteins as well as their involvement in AS and other disorders (53).

## Data Availability Statement

The raw data supporting the conclusions of this article are available through www.proteindiffraction.org (doi: 10.18430/m33czf). The atomic coordinates and structure amplitudes have been deposited in the Protein Data Bank (PDB entry 3CZF).

## Author Contributions

CR performed protein purification and crystallization. BL performed all crystallographic procedures, prepared figures, and wrote the manuscript. BU-Z and AZ conceived the study and wrote the manuscript.

### Conflict of Interest

AZ is the owner of Ziegler Biosolutions. The remaining authors declare that the research was conducted in the absence of any commercial or financial relationships that could be construed as a potential conflict of interest.
